# Creep and Shrinkage Properties of Nano-SiO_2_-Modified Recycled Aggregate Concrete

**DOI:** 10.3390/ma17081904

**Published:** 2024-04-19

**Authors:** Yingwu Zhou, Jiahao Zhuang, Wenwei Lin, Wenzhuo Xu, Rui Hu

**Affiliations:** Guangdong Provincial Key Laboratory of Durability for Marine Civil Engineering, Shenzhen University, Shenzhen 518060, China; ywzhou@szu.edu.cn (Y.Z.); 2110474047@email.szu.edu.cn (J.Z.); 2150471009@email.szu.edu.cn (W.L.); 2100471029@email.szu.edu.cn (W.X.)

**Keywords:** recycled aggregate concrete, nano-SiO_2_, creep and shrinkage, finite element

## Abstract

The poor performance of recycled concrete aggregate (RCA) leads to greater creep in recycled aggregate concrete (RAC) compared to natural aggregate concrete (NAC). To enhance the quality of RCA, this paper utilizes a 2% concentration of a nano-SiO_2_ (NS) solution for pre-soaking RCA. This study aims to replace natural aggregate (NA) with NS-modified recycled aggregate (SRCA) and investigate the creep and shrinkage properties of NS-modified recycled aggregate concrete (SRAC) at various SRCA replacement rates. Subsequently, the creep and shrinkage strains of NAC, SRAC, and RAC are simulated using the finite element method. Finally, a comparative analysis is conducted with the predicted creep and shrinkage strains from CEB-FIP, ACI, B3, and GL2000 models. The experimental results indicate that the creep and shrinkage deformation of SRAC increases with the SRCA replacement rate. Compared to NAC, the creep and shrinkage deformation of SRAC at replacement rates of 30%, 50%, 70%, and 100% increased by 2%, 7%, 13%, and 30%, respectively. However, when 100% of the natural aggregate is replaced with SRCA, the creep and shrinkage deformation decreases by 7% compared to RAC. Moreover, the CEB-FIP and ACI models can predict the creep and shrinkage deformation of concrete reasonably well.

## 1. Introduction

Rapid urbanization and industrialization have led to a tremendous demand for concrete products, particularly in developing countries [1]. The large-scale production of concrete is expected to double the depletion of global NA resources by 2040 [2] and also emit significant amounts of carbon dioxide, posing a substantial threat to the ecological environment. Statistics [3] show that the construction industry consumes 40% of the world’s raw materials, generates about 40% of waste, and is responsible for approximately 25% of carbon dioxide emissions. Besides the use of natural resources, the management of construction and demolition waste (CDW) is a major challenge [4,5]. Currently, most CDW is transported to the outskirts for landfilling. This practice not only increases transportation costs but also occupies extensive land resources and may even pollute the soil and water quality. Therefore, to avoid the over-exploitation of natural resources and in response to the call for green and sustainable development, it is essential to establish systematic and scientific methods for the recycling of CDW [6].

For the recycling and utilization of CDW, the approach of most scholars is to process it into RAC. The specific method for RAC involves crushing, screening, and washing CDW to obtain RCA, which is then used to replace NA in the production of recycled concrete. According to relevant studies, the quality of RAC is significantly inferior to that of NAC, characterized by a lower modulus of elasticity [7,8] and higher creep and shrinkage [9,10,11], among others. Therefore, to enable the wide application of RCA in practical engineering, it is necessary to analyze and study its mechanical performance. This is a multi-dimensional process that requires the investigation of various mutually dependent properties. This research focuses particularly on the phenomenon of creep.

Concrete creep refers to the phenomenon whereby the deformation of concrete components under long-term constant load continuously increases over time. Currently, research on the creep of RAC by related scholars is still in its initial stages, with most studies only considering the impact of the RCA replacement rate on RAC creep and comparing it with NAC creep. Research by Xiao et al. [12] found that the creep of RAC is 20–60% higher than that of NAC and increases with the content of RCA. Hansen and others [13] claim that the creep of RAC is 40–80% higher than NAC due to the presence of an old cement mortar layer on the surface of RCA. Given that the creep of RAC is significantly greater than that of NAC, many scholars have started to adopt various methods to reduce the creep of RAC. Kou et al. [14] have managed to decrease the creep of RAC by lowering the water-to-cement ratio of the concrete, also indicating that the creep of RAC increases with the substitution rate of RCA. Furthermore, research suggests that employing traditional methods for an RAC mix design is a primary reason for its low modulus of elasticity and excessive creep. The literature [9] hypothesizes that due to the presence of a substantial amount of old mortar on the RCA surface, directly replacing NA with RCA in equal proportions effectively reduces the aggregate content while increasing the total mortar (new and old mortar) content in concrete. Therefore, to verify this hypothesis, Fathifazl and others [15] developed a new mix design method for preparing RAC. This novel method is termed the Equivalent Mortar Volume (EMV) method, with detailed steps provided by Fathifazl and colleagues [15]. Fathifazl’s research findings confirmed that the EMV method can produce higher quality RAC at the same strength level.

**Figure 1:**
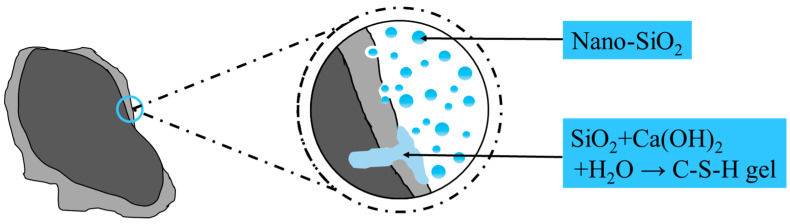
Strengthening mechanism of nano-SiO_2_-modified recycled aggregate.

However, Luo [16] and Ye [17], among others, argue that the creep of concrete is related to the quality of the recycled aggregate. The better the quality of RCA, the smaller the negative impact on its creep properties. Apparent density and water absorption are two primary factors in assessing the quality of RCA. There are numerous methods to improve the quality of RCA, such as mechanical grinding [18], thermal grinding [19], acidic solution soaking for decomposition [20], and polymer emulsion soaking [21], among others. Although these methods can enhance the properties of RCA, most of them have drawbacks like high energy consumption, complex operation, and stringent equipment requirements. However, pre-soaking RCA in a nano- SiO_2_ (NS) solution is an effective modification technique. Research [22,23] has found that NS, due to its large specific surface area and high pozzolanic activity, can accelerate the reaction with a cement hydration product, CH, to form C-S-H gel, filling the voids in the old mortar and thus forming a more compact interfacial transition zone (ITZ) (Figure 1).

Upon reviewing the existing literature, it was found that under the same conditions, the creep strain of RAC is significantly greater than that of NAC. Furthermore, in our previous research [24], we found that the modification effect of a 2% concentration of NS solution is comparable to that of higher concentrations (5%, 10%, and 30%). Therefore, in this paper, we have decided to use a 2% concentration of NS solution to modify RCA to prepare NS-modified recycled aggregate concrete (SRAC), aiming to reduce the creep strain of recycled concrete. The primary objective of this study is to investigate the creep and shrinkage properties of NAC, RAC, and SRAC through experimental research and to analyze the deformation patterns of SRAC at different replacement rates. Moreover, and more importantly, this study aims to identify an optimal replacement rate for NS-modified recycled aggregate (SRCA) that makes the deformation capacity of SRAC comparable to that of NAC, thereby providing feasibility for the use of SRAC in subsequent practical engineering applications. Following this, based on the calculation formulas of the CEB-FIP [25] prediction model, the creep and shrinkage strains of NAC, SRAC, and SRAC are simulated in ABAQUS 2020 software. The purpose of these simulations is to verify the feasibility of using ABAQUS for the calculation of concrete creep and shrinkage, while also offering assistance in predicting the long-term creep strain of concrete. Finally, the creep and shrinkage curves are predicted according to the CEB-FIP MC2010 [25], ACI 209R [26], B3 [27], and GL2000 [28] models, and a comparative analysis with experimental values is conducted.

## 2. Experimental Program

### 2.1. Materials

**Figure 2:**
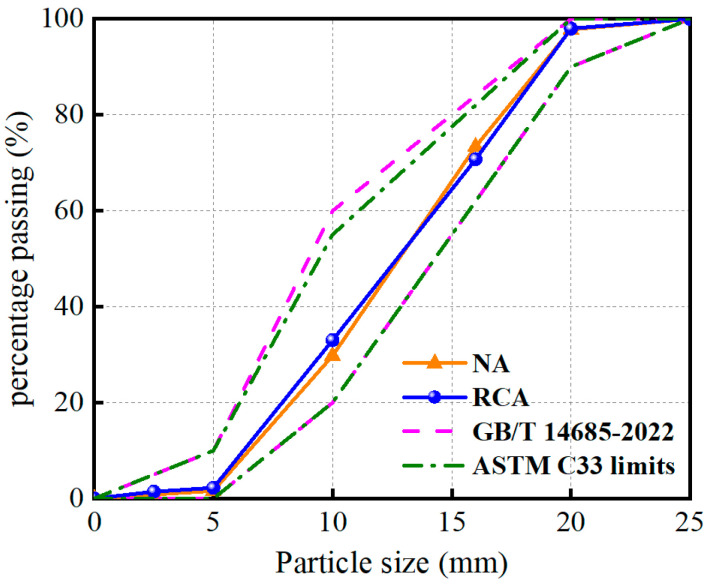
Gradation curve for coarse aggregate.

All concrete mixtures in this study utilized P.O 42.5 Ordinary Portland Cement. The fine aggregate is derived from local natural river sand. The coarse aggregate consists of NA and RCA, with NA coming from local natural granite in Shenzhen, China, and RCA originating from a CDW crushing plant in Shenzhen, China. Although RCA resembled NA in appearance, its apparent density was lower, and the water absorption rate was higher due to the presence of old cement mortar on its surface. The physical properties of NA and RCA are shown in Table 1. In addition, the particle size of the coarse aggregates was 5–20 mm with a continuous gradation. As illustrated in Figure 2, the gradation curves of both NA and RCA conformed to the standards of GB/T 14685-2022 [29] and ASTM C33:2016 [30]. The NS solution was sourced from Zhejiang Yuda Chemical Co., Ltd. in Shaoxing, China, with a mass fraction of 30%, and its main parameters are listed in Table 2.

### 2.2. Modification Process of RCA

**Figure 3:**
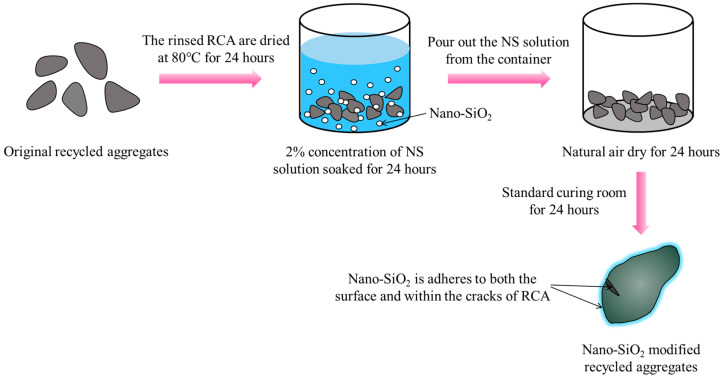
The modification process of RCA.

This paper employs a 2% concentration of NS solution to modify RCA, aiming to achieve higher quality RCA (SRCA). The detailed modification process is illustrated in Figure 3. Firstly, the washed RCA was subjected to drying in an oven at 80 °C for a duration of 24 h. Subsequently, the dried RCA was immersed in a 2% concentration NS solution for 24 h. Upon completion of the soaking period, the RCA was extracted from the solution and allowed to naturally air dry in the laboratory environment for a period of 24 h. Finally, the RCA was transferred to a standard curing room with a relative humidity of 95% and a temperature of 20 °C for 24 h of curing. Compared to RCA, the performance of the recycled aggregate modified with a 2% concentration of NS solution improved, with its water absorption and crushing index decreasing by 18.2% and 20.1%, respectively (Table 1).

### 2.3. Mix Proportion and Details of Specimens

In this study, the water-to-cement (*w*/*c*) ratio for all concrete mixes was 0.36, with detailed mix proportions presented in Table 3. To avoid the impact of varying initial moisture content in the aggregates, we dried the aggregates before casting the concrete to ensure the initial moisture content was consistent across all aggregates [31,32,33]. The naming of concrete specimens is based on a format of Letter1-Letter2-Number. For example, in ‘C-S-30’, ‘C’ stands for creep, ‘S’ stands for SRCA, and ‘30’ indicates an aggregate replacement rate of 30%. To investigate the effect of different replacement rates of recycled coarse aggregate on the creep of recycled concrete, two control groups were designed: NAC (C-N-100) and RAC (C-R-100). By replacing 30%, 50%, 70%, and 100% of NA with an equal volume of SRCA, the creep data of SRAC were obtained and compared with that of NAC. Additionally, this study also compared the recycled concrete of 100% SRCA with the unmodified RAC, analyzing the impact of modified versus unmodified recycled aggregate on creep.

### 2.4. Specimen Preparation and Creep Test Setup

In this experiment, a two-stage mixing method was employed for concrete preparation. Initially, coarse aggregates, sand, and half of the water were mixed together for 2 min to ensure thorough water absorption by the aggregates. Then, cement, admixtures, and the remaining half of the water were combined and mixed for at least 3 min. Subsequently, the mixture was leveled and compacted on a vibrating table. After casting, the molds were removed after 24 h, and then the specimens were cured in a standard curing room at a relative humidity of 95% and a temperature of 20 °C for 28 days. Following these steps, six standard cylindrical specimens of 150 × 300 mm were cast for each mix proportion. Of these, three cylinders were used to determine the concrete’s axial compressive strength and modulus of elasticity, while the other three were utilized for creep testing.

**Figure 4:**
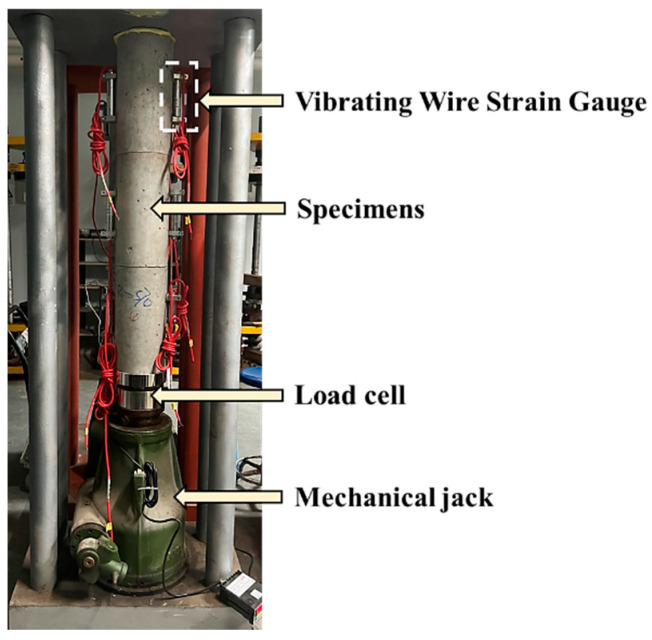
Creep test device.

The creep test was conducted in reference to the steps outlined in ASTM C512/C512M-15 [34]. After the curing period, the cylindrical specimens were transferred to a controlled environment room maintained at 22 ± 2 °C and 55 ± 5% RH for the creep test. Figure 4 shows the loading apparatus for the concrete creep test. As seen in Figure 4, three stacked cylindrical specimens could be tested simultaneously within a rigid frame, with the load applied through an adjustable mechanical jack. Additionally, two vibrating wire strain gauges were placed opposite each other on both sides of each specimen to measure the strain in the concrete. Before applying the initial load, the axial compressive strength of RAC was tested to determine the load to be applied. Ultimately, we determined that all specimens in the creep test were to be subjected to the same load, which was 35% of the axial compressive strength of the RAC [10], equivalent to 11.6 MPa.

### 2.5. Strain Calculation

At any given time, *t*, the total strain of concrete, *ԑ_Total_*(*t*), is calculated as the average value of the data from vibrating wire strain gauges on both sides. The formula for calculating the vibrating wire strain gauge in this paper is as follows:

(1) $$\varepsilon_{Total}\left(t\right)=K\times \left(F_{0}^{2}-T_{t}^{2}\right)$$

where *ԑ_Total_*(*t*) represents the total strain of concrete at any given time, *t*; *K* is the sensitivity of the vibrating wire strain gauge; *F*_0_ is the initial vibrating wire frequency; and *F_t_* is the vibrating wire frequency at time *t*.

The total strain of concrete, *ԑ_Total_*, includes instantaneous elastic strain *ԑ_e_*, creep strain *ԑ_c_*, and shrinkage strain *ԑ_sh_*, as shown in Equation (2).

(2) $$\varepsilon_{Total}\left(t\right)=\varepsilon_{e}\left(t_{0}\right)+\varepsilon_{c}\left(t\right)+\varepsilon_{sh}\left(t\right)$$

## 3. Results and Discussion

### 3.1. Creep and Shrinkage

**Figure 5:**
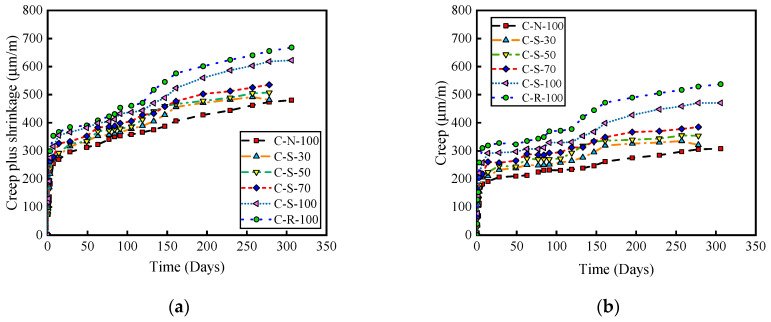
The curve of creep and shrinkage deformation over time. (**a**) Creep plus shrinkage. (**b**) Creep.

Throughout the experiment, the instantaneous strain of all concrete specimens was entirely elastic, with specific experimental values shown in Table 4. As observed in Table 4, the magnitude of ԑe is ranked as follows: NAC < SRAC < RAC. This phenomenon is due to the reduction in concrete’s modulus of elasticity as the SRCA replacement rate increases. Figure 5 displays the curves of creep and shrinkage deformation over time for NAC, SRAC, and RAC. The creep and shrinkage deformation are obtained by subtracting the instantaneous elastic strain from the total strain of the concrete. It can be seen that all curves share a similar shape, with NAC having the smallest creep and shrinkage deformation and RAC the largest. At 278 days, the creep and shrinkage deformation of SRAC with a 30% replacement rate is relatively close to that of NAC; the deformation of SRAC with 50% and 70% replacement rates is 7% and 13% greater than NAC, respectively; and the deformation of SRAC with a 100% replacement rate is about 7% lower than that of RAC.

The research findings of Fathifazl et al. [9] discovered that, regardless of the type of aggregate or the mix proportioning method, the methods of CEB [25] and ACI [26] can accurately predict the curve of concrete shrinkage over time. Therefore, this paper adopts the calculation method of CEB to predict the curve of shrinkage over time, with the formula as follows:

(3) $$\varepsilon_{cs}(t,t_{s})=\varepsilon_{cas}(t)+\varepsilon_{cds}(t,t_{s})$$

(4) $$\varepsilon_{cas}\left(t\right)=-\alpha_{as}{\left(\frac{f_{cm}/10}{6+f_{cm}/10}\right)}^{2.5}\times 10^{-6}\times \left[1-\exp(-0.2\times \sqrt{t})\right]$$

(5) $$\varepsilon_{cds}\left(t,t_{s}\right)=\varepsilon_{cds0}(f_{cm})\times \beta_{RH}\times {\left(\frac{t-t_{s}}{0.035\times h^{2}+(t-t_{s})}\right)}^{0.5}$$

(6) $$\varepsilon_{cds0}(f_{cm})=\left[\left(220+110\times \alpha_{ds1}\right)\times \exp(-\alpha_{ds2}\times f_{cm})\right]\times 10^{-6}$$

(7) $$\beta_{RH}=\left\{\begin{array}{l} -1.55\times \left[1-{\left(\frac{RH}{100}\right)}^{3}\right]\;for\;40\leq RH\leq 99\%\cdot \beta_{s1}\\ 0.25forRH\geq 99\%\cdot \beta_{s1} \end{array}\right.$$

(8) $$\beta_{s1}={\left(\frac{35}{f_{cm}}\right)}^{0.1}\leq 1.0$$

where *ԑ_cs_*(*t*,*t_s_*) represents the total shrinkage strain; *ԑ_cas_*(*t*) is the autogenous shrinkage strain; *ԑ_cds_*(*t*,*t_s_*) is the drying shrinkage strain; *ԑ_cds_*_0_(*f_cm_*) is the nominal drying shrinkage coefficient of concrete; α*_as_*, α*_ds_*_1_, and α*_ds_*_2_ are parameters depending on the cement quality and are taken as 700, 4, and 0.012, respectively, in this study; *β_RH_*(*h*) is the coefficient of environmental humidity’s impact on drying shrinkage; and *β_as_*(*t*) is the function of autogenous shrinkage over time. *f_cm_* is the average 28 d compressive strength of concrete. *RH* is relative humidity (60%). *β_s_*_1_ is the coefficient considering the effect of self-dehydration in high-performance concrete.

The creep data (Figure 5b) are obtained by subtracting the shrinkage values predicted by the CEB formula from the data in Figure 5a. It is observed that the creep values of SRAC with replacement rates of 30%, 50%, 70%, and 100% are, respectively 5%, 16%, 26%, and 54% higher than those of the control group NAC. However, compared to the control group RAC, the creep value of SRAC with a 100% replacement rate decreases by 11%. This indicates that using modified SRCA to prepare concrete can effectively reduce the creep strain of recycled concrete.

### 3.2. Creep Coefficient

**Figure 6:**
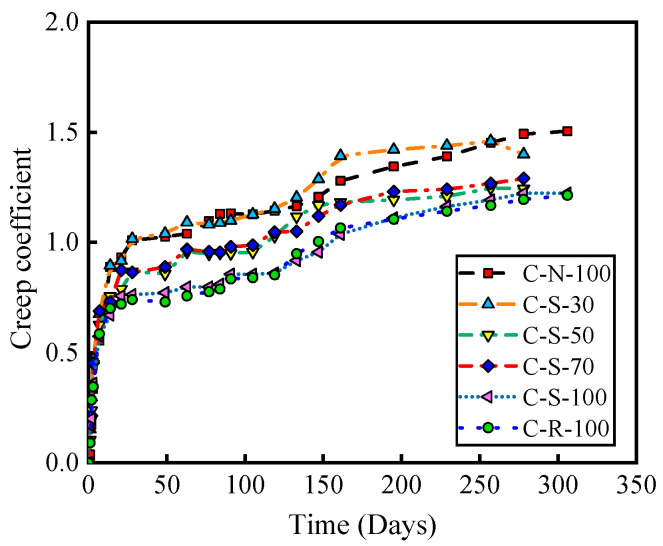
The relationship between creep coefficient and age of loading.

The creep coefficient is defined as the ratio of the concrete’s creep strain at a given moment to the elastic strain at the moment of loading under constant stress. It is an important physical quantity that describes the deformation characteristics of a material. The higher the value of the creep coefficient, the stronger the concrete’s capacity to deform under stress, indicating better resistance to deformation. Conversely, a lower value signifies poorer resistance to deformation. Figure 6 illustrates the curves of the creep coefficient over time for NAC, SRAC, and RAC. The graph shows that the creep coefficient of SRAC with a 30% replacement rate is close to that of NAC, indicating that replacing 30% of NA with SRCA has a minimal impact on the concrete’s resistance to deformation. The curves of the creep coefficient over time for SRAC with 50% and 70% replacement rates almost overlap, suggesting similar resistance to deformation. The creep coefficient of SRAC with a 100% replacement rate is not significantly different from that of RAC.

### 3.3. The Impact of Different SRCA Replacement Rates on Concrete Creep and Shrinkage Deformation

**Figure 7:**
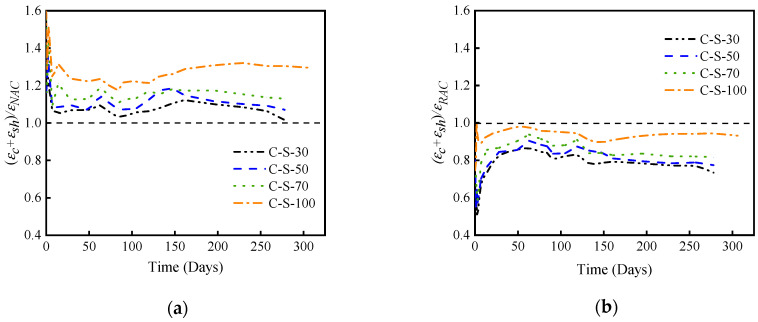
Comparing the deformation of SRAC with two control groups. (**a**) Comparing the deformation of SRAC and NAC. (**b**) Comparing the deformation of SRAC and RAC.

Figure 7a shows the deformation relationship between SRAC with different SRCA substitution rates and NAC. At 278 days of loading, the deformation of SRAC with substitution rates of 30%, 50%, 70%, and 100% increased by 2%, 7%, 13%, and 30%, respectively, compared to NAC. The test curves for 30% and 50% substitution rates show a significant downward trend, approaching that of NAC. As the age of loading progresses, it is likely that the deformation of these two test groups will become comparable to NAC. Figure 7b illustrates the deformation relationship between SRAC with different SRCA substitution rates and RAC. At 278 days of loading, compared to RAC, the deformation of SRAC with substitution rates of 30%, 50%, 70%, and 100% decreased by 7%, 18%, 23%, and 27%, respectively. Although the decrease in deformation for 100% SRAC compared to RAC is not very pronounced, it still reduces the deformation of the concrete components to some extent.

## 4. Numerical Simulation

This paper establishes a two-dimensional concrete model in ABAQUS 2020 software based on the CEB-FIP prediction model and carries out simulations of concrete creep. Finally, the experimental creep and shrinkage curves are compared and analyzed against the simulated curves.

### 4.1. Finite Element Modeling

**Figure 8:**
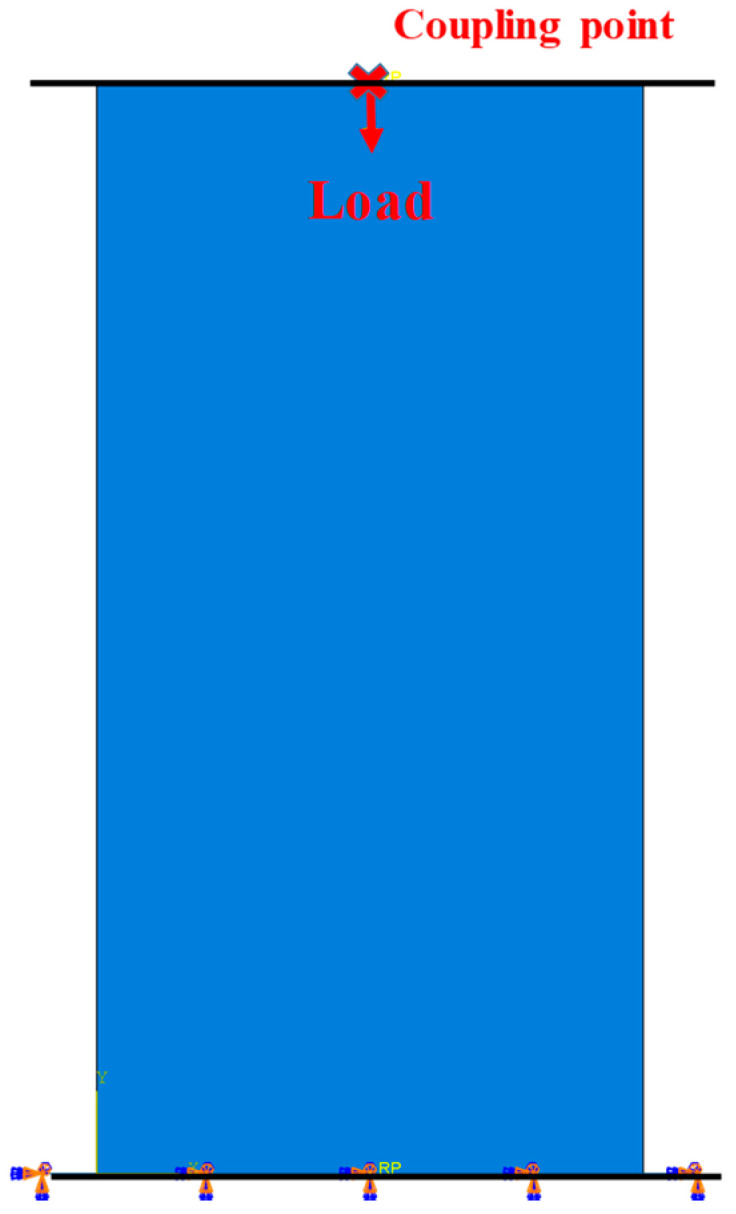
Two-dimensional geometric model.

This study constructs a two-dimensional geometric model (Figure 8) based on the actual dimensions of the concrete specimens used in the experiments and selects the 4-node bilinear plane stress quadrilateral element (CPS4R) as the mesh computation unit, totaling 1800 elements. The simulation in this research is carried out by applying loads through a force-loading method. Two pads are set on the *y*-axis of the concrete, with the lower pad being fully fixed and the upper pad subjected to a constant force. To prevent uneven force distribution on the top of the concrete specimen, a reference point is coupled at the top, and a concentrated force is applied to this reference point to distribute the force evenly across the entire model.

For the sake of simplification in calculations, this article defines the entire concrete specimen as a homogeneous body with a density of 2.1 g/cm^3^. Moreover, in the material definition of the homogenous body, the Concrete Damage Plasticity (CDP) model, dilation, elasticity, and user-defined fields are included. The parameters of the CDP model primarily concern the Yield Stress and Inelastic Strain of concrete under uniaxial stress conditions, where the Inelastic Strain is calculated based on the constitutive relations of concrete [35]. Furthermore, when imposing loads that generate deformations greater than the threshold that defines the elastic-linear regime, this study considers material damage by introducing a damage factor [36,37]. The larger the damage factor, the more severe the material damage, with the value ranging from 0 to 1. The calculation method for the damage factors shown in Formula (9) [38].

(9) $$d_{k}=\frac{\left(1-\beta \right)\varepsilon^{in}E_{0}}{\alpha_{k}+(1-\beta )\varepsilon^{in}E_{0}},\left(k=t,c\right)$$

where *d_k_* represents the damage factor; *α_k_* denotes the parameters of the descending segment of the stress–strain curve; *t* and *c*, respectively, stand for tension and compression; *β* is the proportionality coefficient between plastic strain and inelastic strain, taken as 0.95 in tension and 0.7 in compression; *ԑ^in^* is the strain during the inelastic phase of the concrete; and *E*_0_ stands for the initial elastic modulus.

The material parameters used in the modeling are detailed in Table 5 as follows. The remaining parameters of concrete are as follows: the dilation angle is 40°, plastic eccentricity is 0.1, the ratio of biaxial to uniaxial compressive strength is 1.16, and the concrete yield shape is parameter is 0.667.

### 4.2. ABAQUS Subroutines

**Figure 9:**
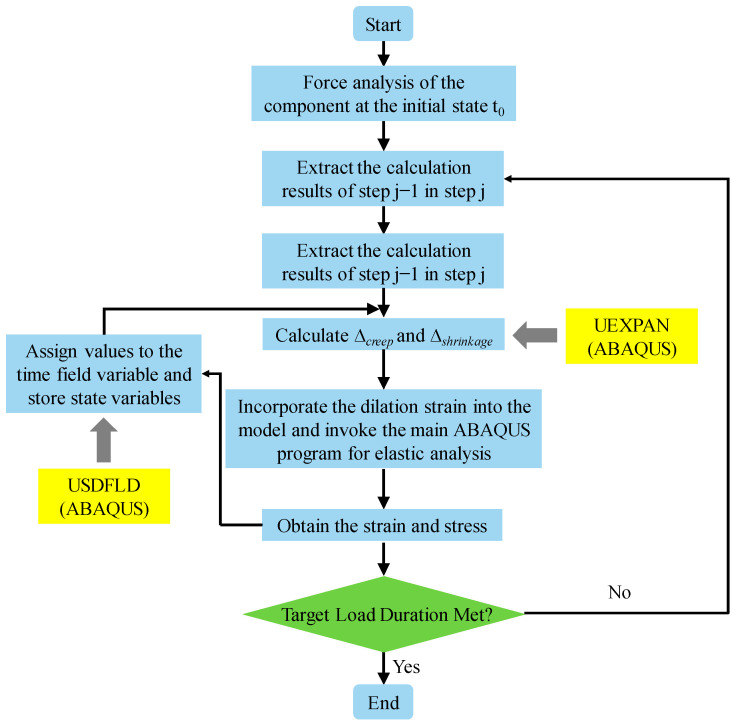
The subroutine flowchart.

This paper conducts secondary development on the Fortran program [39], which primarily includes two subroutines: USDFLD and UEXPAN. The USDFLD subroutine is used to control a parameter of the material by introducing a field variable, thereby defining the complex material characteristics that change with the field variable. This subroutine is utilized to define the time-varying elastic modulus of concrete in this study. Additionally, the UEXPAN subroutine is the core part of the program for calculating creep and shrinkage, used to compute the expansion strain caused by state variables. The methods for calculating creep and shrinkage are incorporated into the UEXPAN subroutine to calculate the creep strain and shrinkage strain of concrete separately, and the total strain is obtained by summing the two. The subroutine flowchart is as shown in Figure 9.

ABAQUS calculates the creep and shrinkage of concrete by invoking the USDFLD and UEXPAN subroutines. This paper adopts a time interval of 2 days to ensure the accuracy of the calculations.

### 4.3. Verification of the Model

**Figure 10:**
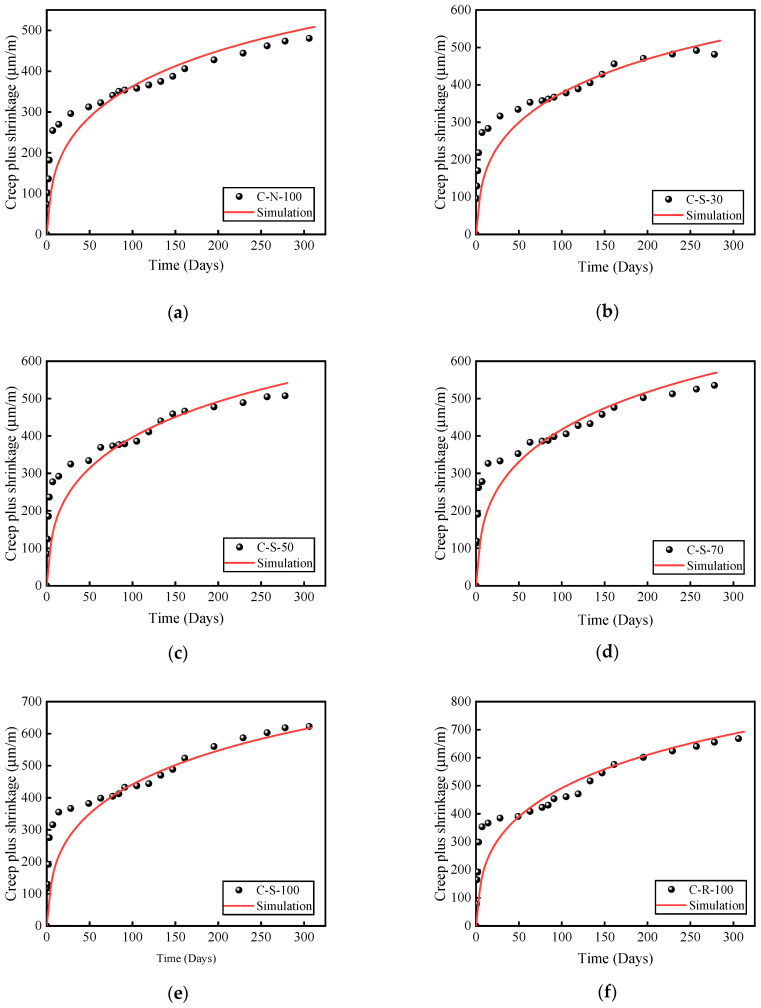
Comparison of experimental and simulated curves. (**a**) C-N-100. (**b**) C-S-30. (**c**) C-S-50. (**d**) C-S-70. (**e**) C-S-100. (**f**) C-R-100.

Figure 10 displays a comparison between the experimental and simulated curves for different types of aggregates. As seen from Figure 10, there is a significant discrepancy between the experimental and simulation values within the first 28 days, but the results tend to agree closely after 28 days. Within the initial 28 days, the results calculated by ABAQUS are smaller, largely due to the fact that the superposition principle can introduce certain errors in the calculation of creep and shrinkage in early-age concrete, and the experiment itself is greatly influenced by environmental factors and the grading of concrete. Overall, the ABAQUS modeling approach and parameter settings employed in this study are capable of simulating the creep and shrinkage of concrete well, thereby verifying the feasibility of using ABAQUS for creep and shrinkage calculations.

## 5. Comparison of Results with Existing Predictive Models

**Figure 11:**
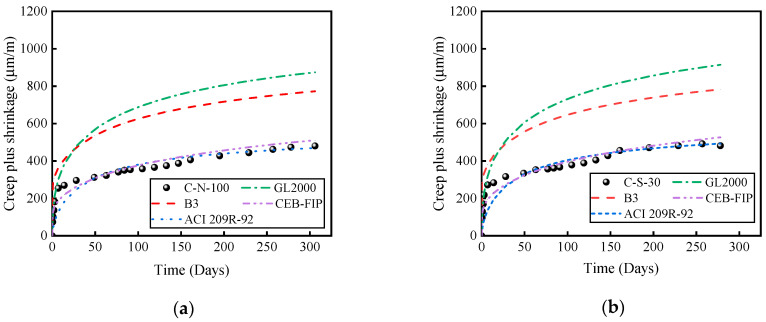

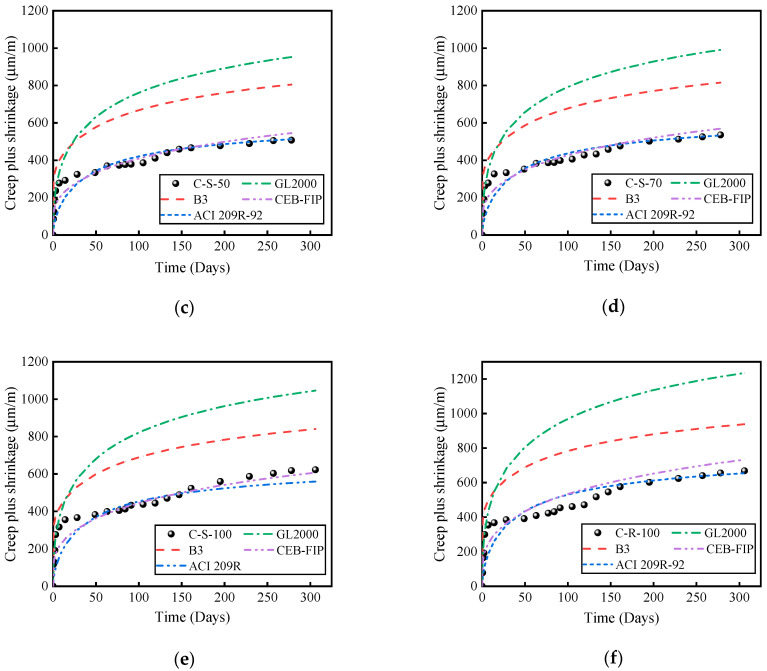
Comparison of experimental results with predictive outcomes. (**a**) C-N-100. (**b**) C-S-30. (**c**) C-S-50. (**d**) C-S-70. (**e**) C-S-100. (**f**) C-R-100.

According to the literature, the most commonly used models for predicting concrete creep and shrinkage include CEB-FIP MC2010 [25], ACI 209R-92 [26], B3 [27], and GL2000 models [28] (Table 6). Figure 11 presents a comparative analysis between the experimental results and the predictions of these four models. It is evident that the CEB-FIP MC2010 and ACI 209R-92 models provide predictions of creep and shrinkage strains over time that closely align with the experimental curves. However, the B3 and GL2000 models significantly overestimate the experimental values of deformation. This observation aligns with the findings of Fathifazl [9], indicating that regardless of the aggregate type, the CEB and ACI models can accurately predict the time-dependent deformation curves.

## 6. Conclusions

This paper, through a series of experiments, thoroughly investigates the creep and shrinkage properties of SRAC at different substitution rates of SRCA. Based on the results of the experiments previously discussed, the following conclusions can be drawn:

(a) Using NS-modified SRCA to replace unmodified RCA can reduce the creep of recycled aggregate concrete. Compared to RAC, the creep and shrinkage deformations of SRAC with substitution rates of 30%, 50%, 70%, and 100% are reduced by 7%, 18%, 23%, and 27%, respectively.
(b) Based on the creep coefficient, it is possible to conclude that the deformation resistance of SRAC with a 30% substitution rate is comparable to that of NAC.
(c) Based on the prediction model of CEB-FIP MC2010, the curves simulated in ABAQUS in this study generally match the experimental curves. However, before 28 days, the deformation values calculated by ABAQUS are smaller.
(d) Comparing the experimental results with the predictions of four models, it was found that the results predicted by the empirical methods of CEB-FIP and ACI align very closely, while the predictions from the B3 and GL2000 models are larger, indicating that the calculations from these two models are overly conservative.

## Figures and Tables

**Table 1:** Physical characteristics of NA, RCA, and SRCA.

Aggregate Category	Apparent Density (kg/m^3^)	Water Absorption (%)	Crushing Index (%)
NA	2652	1.40	11.28
RCA	2564	6.27	15.42
SRCA	2588	5.13	12.32

**Table 2:** Physical attributes of NS solution.

Particle Size (nm)	Color	Nano-SiO_2_ Content (%)	PH	Density (g/cm^3^)	NaO_2_ Content (%)
12	White	>99.9	10.3	1.2	0.38

**Table 3:** Mix proportions of concrete (kg/m^3^).

Specimen	SRCA Content (%)	NA	SRCA	RCA	Water	Cement	Sand	SP
C-N-100	0	1032.0	0	0	180.0	500.0	688.0	0.03
C-S-30	30	722.4	309.6	0	180.0	500.0	688.0	0.03
C-S-50	50	516.0	516.0	0	180.0	500.0	688.0	0.03
C-S-70	70	309.6	722.4	0	180.0	500.0	688.0	0.03
C-S-100	100	0	1032.0	0	180.0	500.0	688.0	0.03
C-R-100	0	0	0	1032.0	180.0	500.0	688.0	0.03

**Table 4:** Axial compressive strength, elastic modulus, and *ԑ_e_* for each specimen.

Properties	C-N-100	C-S-30	C-S-50	C-S-70	C-S-100	C-R-100
$\sigma_{cp}$ (MPa)	50.06	46.86	44.10	42.92	41.59	33.18
*Ε* (Mpa)	37,141	33,906	32,028	30,337	28,905	23,391
*ԑ_e_* (μm/m)	204.6	229.2	284.4	298.1	384.7	442.5

**Table 5:** Material parameters in modeling.

Parameter	C-N-100	C-S-30	C-S-50	C-S-70	C-S-100	C-R-100
*f_cm_* (mPa)	50.06	46.86	44.10	42.92	41.59	33.18
*Ε* (mPa)	37,141	33,906	32,028	30,337	28,905	23,391
*ρ* (g/cm^3^)	2.1	2.1	2.1	2.1	2.1	2.1
*ν*	0.2	0.2	0.2	0.2	0.2	0.2

Note: *f_cm_* = axial compressive strength; E = elasticity modulus; *ρ* = density; ν = Poisson’s ratio.

**Table 6:** The creep and shrinkage formulas of four models.

Model	Creep	Shrinkage
CEB-FIP MC2010 [25]	$\varphi (t,t_{0})=\varphi_{0}\beta_{c}(t-t_{0})$	$\varepsilon_{sh}(t,t_{c})=\varepsilon_{ca}(t)+\varepsilon_{cd}(t,t_{c})$
ACI 209R-92 [26]	$\varphi (t,t_{0})=\frac{{\left(t-t_{0}\right)}^{0.6}}{10+{\left(t-t_{0}\right)}^{0.6}}\varphi (\infty )$	$\varepsilon_{sh}(t,t_{c})=\frac{{\left(t-t_{c}\right)}^{\alpha }}{f+{\left(t-t_{c}\right)}^{\alpha }}\varepsilon_{sh\infty }$
B3 [27]	$J(t,t_{0})=C_{0}(t,t_{0})+C_{d}(t,t_{0},t_{c})$	$\varepsilon_{sh}(t,t_{c})=-\varepsilon_{sh\infty }k_{h}S(t-t_{c})$
GL2000 [28]	$\begin{array}{l} \varphi (t,t_{0})=\varphi (t_{c})[2\times (\frac{{\left(t-t_{0}\right)}^{0.3}}{{\left(t-t_{0}\right)}^{0.3}+14})+\sqrt{\frac{7}{t_{0}}}\times \sqrt{\frac{t-t_{0}}{t-t_{0}+7}}\\ +2.5\times (1-1.086h^{2})\times \sqrt{\frac{t-t_{0}}{t-t_{0}+0.5{\left(V/S\right)}^{2}}}] \end{array}$	$\varepsilon_{sh}(t,t_{c})=\varepsilon_{shu}\beta (h)\beta (t-t_{c})$

Note: *φ* is the creep coefficient; *J* is the creep compliance; *φ*_0_ is the nominal creep coefficient; *β_c_* is the function of creep over time; *t* is the age of concrete at the time of calculation (days); *t*_0_ is the age of concrete at loading (days); *t_c_* is the age of concrete when drying starts (days); *φ*(∞) is the ultimate creep value; *ε_sh_* is the total shrinkage strain; *ε_ca_* is the autogenous shrinkage strain; *ε_cd_* is the drying shrinkage strain; *ε_sh∞_* is the ultimate shrinkage strain of concrete; *k_h_* is the temperature influence coefficient; *–*(*t − t_c_*) is the function of shrinkage over time; *ε_shu_* is the nominal shrinkage coefficient; *β*(*h*) is the temperature influence correction coefficient; and *β*(*t − t_c_*) is the time-dependent correction coefficient for shrinkage.

## Data Availability

Data are contained within the article.
